# Genome-wide identification of the Q-type C2H2 zinc finger protein gene family and expression analysis under abiotic stress in lotus (*Nelumbo nucifera* G.)

**DOI:** 10.1186/s12864-024-10546-1

**Published:** 2024-06-28

**Authors:** Huan Liu, Yidan Liu, Fangyu Liu, Lihong Zeng, Yingchun Xu, Qijiang Jin, Yanjie Wang

**Affiliations:** 1 Key Laboratory of Landscaping, Ministry of Agriculture and Rural Affairs, Key Laboratory of Biology of Ornamental Plants in East China, College of Horticulture, Jiangsu Province, Nanjing Agricultural University, Weigang No.1, Nanjing, 210095 People’s Republic of China; 2 College of Horticulture, Nanjing Agricultural University, Nanjing, 210095 China

**Keywords:** Aquatic macrophyte, Environmental stresses, Evolution, Gene expression, Transcription factors

## Abstract

**Background:**

Lotus (*Nelumbo nucifera* G.) is an important aquatic plant with high ornamental, economic, cultural and ecological values, but abiotic stresses seriously affect its growth and distribution. Q-type C2H2 zinc finger proteins (ZFPs) play an important role in plant growth development and environmental stress responses. Although the Q-type C2H2 gene family has been identified in some plants, limited reports has been carried out it in lotus.

**Results:**

In this study, we identified 45 Q-type *NnZFP* members in lotus. Based on the phylogenetic tree, these Q-type *NnZFP* gene family members were divided into 4 groups, including C1-1i, C1-2i, C1-3i and C1-4i. Promoter *cis*-acting elements analysis indicated that most Q-type *NnZFP* gene family members in lotus were associated with response to abiotic stresses. Through collinearity analyses, no tandem duplication gene pairs and 14 segmental duplication gene pairs were identified, which showed that duplication events might play a key role in the expansion of the Q-type *NnZFP* gene family. The synteny results suggested that 54 and 28 Q-type *NnZFP* genes were orthologous to Arabidopsis and rice, respectively. The expression patterns of these Q-type *NnZFP* genes revealed that 30 Q-type *NnZFP* genes were expressed in at least one lotus tissue. *Nn5g30550* showed relatively higher expression levels in all tested tissues. 12 genes were randomly selected with at least one gene from each phylogenetic clade, and the expression of these selected genes were confirmed by qRT-PCR (quantitative real-time polymerase chain reaction). The results indicated that Q-type *NnZFP* genes were extensively involved in cadmium, drought, salt and cold stresses responses. Among them, 11 genes responded to at least three different stress treatments, especially *Nn2g12894*, which induced by all four treatments.

**Conclusions:**

These results could increase our understanding of the characterization of the Q-type *NnZFP* gene family and provide relevant information for further functional analysis of Q-type *NnZFP* genes in plant development, and abiotic stress tolerance in lotus.

**Supplementary Information:**

The online version contains supplementary material available at 10.1186/s12864-024-10546-1.

## Background

Plant growth and development has been threatened by various abiotic stresses [1], such as drought, salinity, extreme temperatures, and heavy metals. Transcription factors (TFs) act as critical regulators involved in various biological and environmental stress processes through transcriptional regulation of downstream genes in plants [2]. Zinc finger proteins (ZFPs) are one of the largest families of TFs in plants, and they can self-fold into a "finger" structure by binding to Zn^2+^ [3]. ZFPs can be classified into different categories based on the position and quantity of His and Cys residues, including C4HC3, C3H, C3HC4, C4, C2H2, C2HC, C6, and parallel types [4]. Among them, C2H2 ZFPs consist of two Cys and two His residues that coordinate with a zinc atom, forming a zinc finger structure with a β-sheet and an α-helix[5].

The Q-type ZFP subfamily with “X_2_-C-X_2_-C-X_3_-F-X_3_-QALGGH-X_3_-H”, belongs to the C2H2 ZFPs family [6, 7], and they have been isolated from various plant species, such as petunia [8], Arabidopsis [9], poplar [10], rice [11], wheat [12], and potato [13]. These Q-type ZFPs play vital roles in growth and various abiotic stress responses in plants [14–17]. For example, the expression of *StZFP1* in potato increases under salt and drought stress, and ectopic expression of *StZFP1* in tobacco enhances salt tolerance of transgenic plants [18]. In addition, overexpression of *AtZAT10* improves cold resistance in Arabidopsis [19], and overexpressing *OsZFP245* increases drought tolerance in rice [20]. *PuZFP103* from poplar enhances drought tolerance by regulating the levels of substances such as MDA, EL, proline, and soluble sugars [21]. *AtZAT6* enhances cadmium tolerance in Arabidopsis through specifically binding to the promoter of *GSH1* and regulating glutathione synthesis [22]. However, these studies have mainly focused on model or terrestrial plants, with limited reports in aquatic plants.

Lotus (*Nelumbo nucifera* G.) is a perennial aquatic plant belonging to the Nelumbonaceae family. It is one of the traditional flowers in China with high ornamental, economic, cultural and ecological values, and very popular cultivated worldwide nowadays [23]. In addition, lotus is a basal eudicot plant with numerous monocot characteristics, so it is also an important subject for evolutionary and taxonomic studies [24]. However, adverse environment stresses such as extreme temperatures, salinity, heavy metals and drought stresses greatly affect lotus survival and growth, including leaf yellowing and wilting, petal decayed, and disruption of the redox balance system, photosynthesis system, and cellular structure [25–27]. These studies are mainly limited to phenotype, physiological and biochemical characteristics of lotus responses to environment stresses, but the exact molecular mechanism of stress resistance in lotus remains largely unknown. Therefore, the exploration of stress-responsive genes relate to lotus is of great importance for its broader applications. To date, most of the reports relate to stress-responsive transcription factors of lotus have focused on bHLH [24], WRKY [28], WUSCHEL-related homeobox (WOX) [29], and so on. However, no lotus Q-type ZFP family genes participate in various abiotic stress have yet been identified.

In the current study, we firstly identified Q-type *NnZFPs* according to the genome of lotus, and then analyzed their phylogenetic relationships, sequence features, chromosome distribution, gene duplications and *cis*-regulatory elements. In addition, the expression profiles of these Q-type *NnZFP* genes in various tissues and their responses to abiotic stresses were investigated. These results will provide valuable information in predicting the roles of the Q-type *NnZFP* gene family in lotus. Moreover, the study could lay the foundation for creating resistant resources of lotus through molecular breeding.

## Materials and methods

### Plant materials and treatments

The seeds of lotus Weishanhuhonglian were provided by Weishanhu Hedu Aquatic Flower Breeding Base (Shandong, China). The seeds were germinated in distilled water with the top part of the blunt end pierced. And they were cultured for next 3 weeks at 30 ℃ with a long-day photoperiod of 16 h light/8 h dark, and a light intensity of 12,000 lx. After three weeks, healthy and uniformly growing seedlings were selected for different abiotic stress treatments, including drought stress treatment (20% polyethylene glycol, PEG6000), salt stress treatment (300 mmol/L NaCl), cadmium stress treatment (30 μmol/L CdCl2), and low temperature treatment (4℃). In contrast, seedlings were cultured in distilled water as a control. Five treatment time points (0, 6, 12, 24 and 48 h) were performed. The experiment was performed with three biological replicates. Leaf samples from the treated and control seedlings were collected, frozen in liquid nitrogen, and stored at -80 °C for subsequent RNA extraction.

### Database sources and identification of Q-type *NnZFPs* in lotus

The protein sequences of the Q-type C2H2 ZFP gene family in *Arabidopsis thaliana* L. were downloaded from database (https://www.arabidopsis.org/) according to a published article [1]. All these protein sequences were queried to search the lotus Q-type C2H2 family through local protein blast (BLASTP) against the whole genome sequence database (http://nelumbo.biocloud.net), with an E value ≤ 1^e−5^. After removing redundant sequences, the candidates were further submitted to SMART (http://smart.embl-heidelberg.de/) and the NCBI Conserved Domain Database CDD-search (https://www.ncbi.nlm.nih.gov/) was used to manually screen Q-type C2H2 ZFP members in lotus for further analysis.

### Analysis of phylogenetic, gene structure and conserved composition of Q-type *NnZFPs* in lotus

The number of amino acid, molecular weight and isoelectric point of Q-type *NnZFPs* were analyzed using the online tool Expasy (http://web.expasy.org/computepi/). Subcellular localization was predicted using the WoLF PSORT website (https://wolfpsort.hgc.jp/). The phylogenetic tree was constructed based on the alignment of amino acid sequences for Arabidopsis Q-type ZFPs and lotus Q-type ZFPs members by MEGA11 with the maximum likelihood method and 500 replicate bootstrap tests. The MEME 5.5.0 software (http://meme-suite.org/tools/meme) was used to predict protein conserved motifs, with the following parameters: maximum number of motifs: 10; and the optimum motif widths: 5–150 amino acid residues. The analyses of exon–intron structures of Q-type *NnZFP* genes were carried out by comparing the coding sequences with their corresponding protein sequences. Then the map of phylogenetic tree, conserved motifs, and intron–exon structures of Q-type *NnZFPs* were visualized and merged using TBtools [30].

### Analysis of chromosomal distribution, gene duplication and *cis*-regulatory element

The chromosome distribution information of each Q-type *NnZFP* gene was acquired from the genome annotation data of lotus. Based on TBtools, the chromosomal positions map of Q-type *NnZFP* genes and the relative distances were obtained. MCScanX software was used to analyze the duplication events and synteny of the Q-type ZFPs between lotus and Arabidopsis, and lotus and rice. The resultant microsynteny relationships were further evaluated by CollinearScan set at an Evalue of < 1^e−5^, and the figures were drawn by TBtools [30]. In addition, the ratio of non-synonymous to synonymous nucleotide substitutions (Ka/Ks) was evaluated among duplicated gene pairs to detect the selection mode by Ka/Ks Calculator and ParaAT2.0. The sequences of 2000 bp from the promoter region of these Q-type *NnZFP* genes were extracted using TBtools [30]. Then the PlantCARE database (http://bioinformatics.psb.ugent.be/webtools/plantcare/html/) was used to identify the *cis-*regulatory elements of these Q-type *NnZFPs*. Among them, we chose 8 *cis*-elements that were most related to abiotic stress, hormones and plant growth, including TGTCGG/TGTCTC (AuXREs), MYB-binding sites (MBSs), abscisic acid response elements (ABREs), G-boxes (LREs), CGTCA/TGACG-motif (JAREs), dehydration-responsive element/C-repeat (DRE/CRT), TCA-elements, WRE and WUN-motif. The results were finally visualized using TBtools [30].

### Tissue specific expression of Q-type *NnZFP* genes

Based on genome-wide transcriptome data downloaded from the lotus Genome Database (http://nelumbo.cngb.org/nelumbo/tools/expressionVisualization) [31], the expression profiles of Q-type *NnZFP* genes in various tissues (apical bud, seed coat, cotyledon, root, rhizome internode, immature receptacle, mature receptacle, leaf, immature stamen, mature stamen, petal, petiole, pollinated carpel, unpollinated carpel, rhizome elongation and rhizome apical meristem) from all developmental stages were explored [31]. These data and the IDs of these Q-type *NnZFPs* were uploaded to the “HeatMap” procedure in TBtools [30]. In control dialog, lower and higher levels of transcript accumulation were indicated by red and blue, respectively, and the median level was signaled by white, the expression heatmap was generated subsequently.

### RNA isolation and quantitative real-time PCR

A total of twelve Q-type *NnZFP* genes were randomly selected as candidates and the synthesized primers in Table S1 were designed by primer premier 5. Total RNA were extracted with the RNA extraction kit (Vazyme, NanJing, China). Elimination of genomic DNA contamination and first-strand cDNA synthesis were carried out using the PrimeScript™ RT reagent Kit with gDNA Eraser (Vazyme, NanJing, China). The qRT-PCR analyses of the expression of each gene under different stress conditions were performed using the 2 × Taq Master Mix ChamQ Universal SYBR qPCR Master Mix kit (Vazyme, NanJing, China) with the QuantStudioTM Real-Time PCR system (QuantStudio5, Applied Biosystems, Hammonton, NJ, USA). The programs were performed as the following steps: 95 °C/30 s for pre-denaturation (step 1), 95 °C/10 s for denaturation (step 2), 60 °C/30 s and 95 °C/15 s for primer annealing and extension (step 3), then go to step 2 for 40 circles, and the collection program of melting curve was default in the system. The obtained data were analyzed using the 2^−△△Ct^ method [32], with *NnActin* (LOC104593066) serving as the internal control [33].

### Statistical analysis

All statistical analyses were calculated with SPSS version 18.0 (SPSS Inc., Chicago, IL, USA). Statistical differences between measurements on different treatments were determined by Students *t*-test for significance analysis (*: *p<*0.05; **: *p<*0.01). All measurements were performed with three biological replicates and three technical replicates of each biological replicate, and data were expressed as mean ± standard deviations (SD).

## Results

### Identification and physicochemical properties of Q-type *NnZFPs*

The BLASTP algorithm search was performed against the lotus genome, and then we manually selected the Q-type ZFP members with the specific sequence “X_2_-C-X_2_-C-X_3_-F-X_3_-QALGGH-X_3_-H”. Finally, a total of 45 Q-type ZFPs in lotus were obtained after screening domains and removing redundant genes. Physicochemical analyses of the 45 Q-type *NnZFPs* revealed that they ranged from 145 to 590 amino acids in length. The molecular weights of these proteins were varied from 16,179.64 to 65,646.06, and their isoelectric point (pI) ranged from 5.02 to 9.27. Moreover, subcellular localization prediction indicated that the majority of Q-type *NnZFP* members (34/45, 75.6%) were predicted to be localized in the cell nucleus, while only 11 members showed potential localization in other cellular compartments, including peroxisomes, extracellular matrix, chloroplasts, endoplasmic reticulum, plasma membrane, and cytoplasm (Table S2).

### Phylogenetic analyses and classification of Q-type *NnZFPs*

**Fig. 1:**
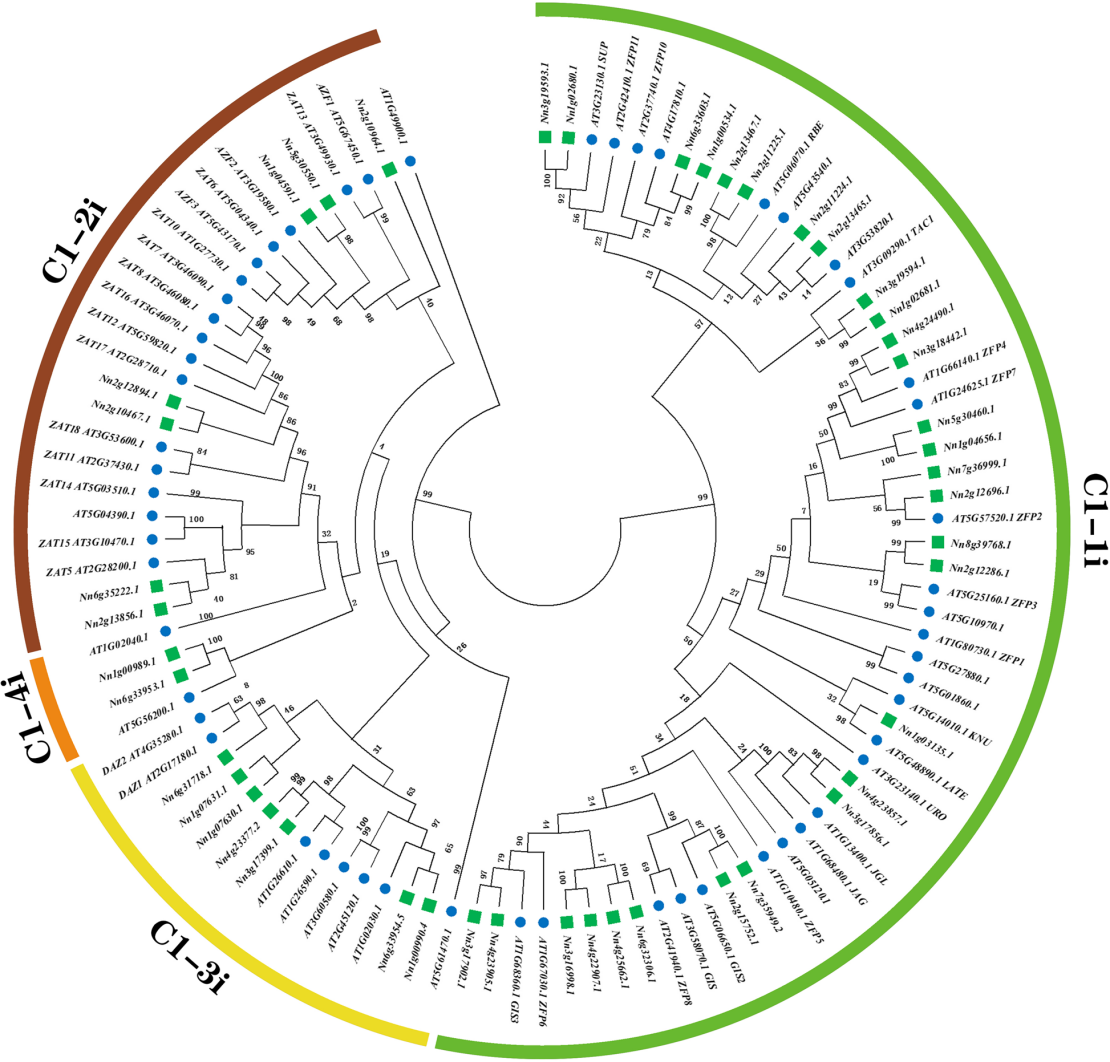
Phylogenetic analysis and classification of Q-type *NnZFPs* and Q-type *AtZFPs*. The phylogenetic tree represents the relationship between 45 Q-type ZFP genes of lotus and 56 Q-type ZFP genes of Arabidopsis. All genes were clustered into four clades

To clearly investigate evolutionary relationships, a phylogenetic tree was constructed based on the alignment of amino acid sequences for 56 Arabidopsis Q-type ZFPs and 45 lotus Q-type ZFPs members by MEGA11 with the maximum likelihood method and 500 replicate bootstrap tests. According to the phylogenetic tree (Fig. 1), the Q-type ZFP subfamily were divided into four clades, namely C1-1i, C1-2i, C1-3i, and C1-4i. Twenty-nine *NnZFPs* and twenty-eight *AtZFPs* were assigned to the C1-1i group, seven *NnZFPs* and eighteen *AtZFPs* belonged to the C1-2i group, seven *NnZFPs* and eight *AtZFPs* were grouped into the C1-3i group, and C1-4i group possessed two *NnZFPs* and two *AtZFPs*.

### Gene structure and motif composition analyses of the Q-type *NnZFPs*

**Fig. 2:**
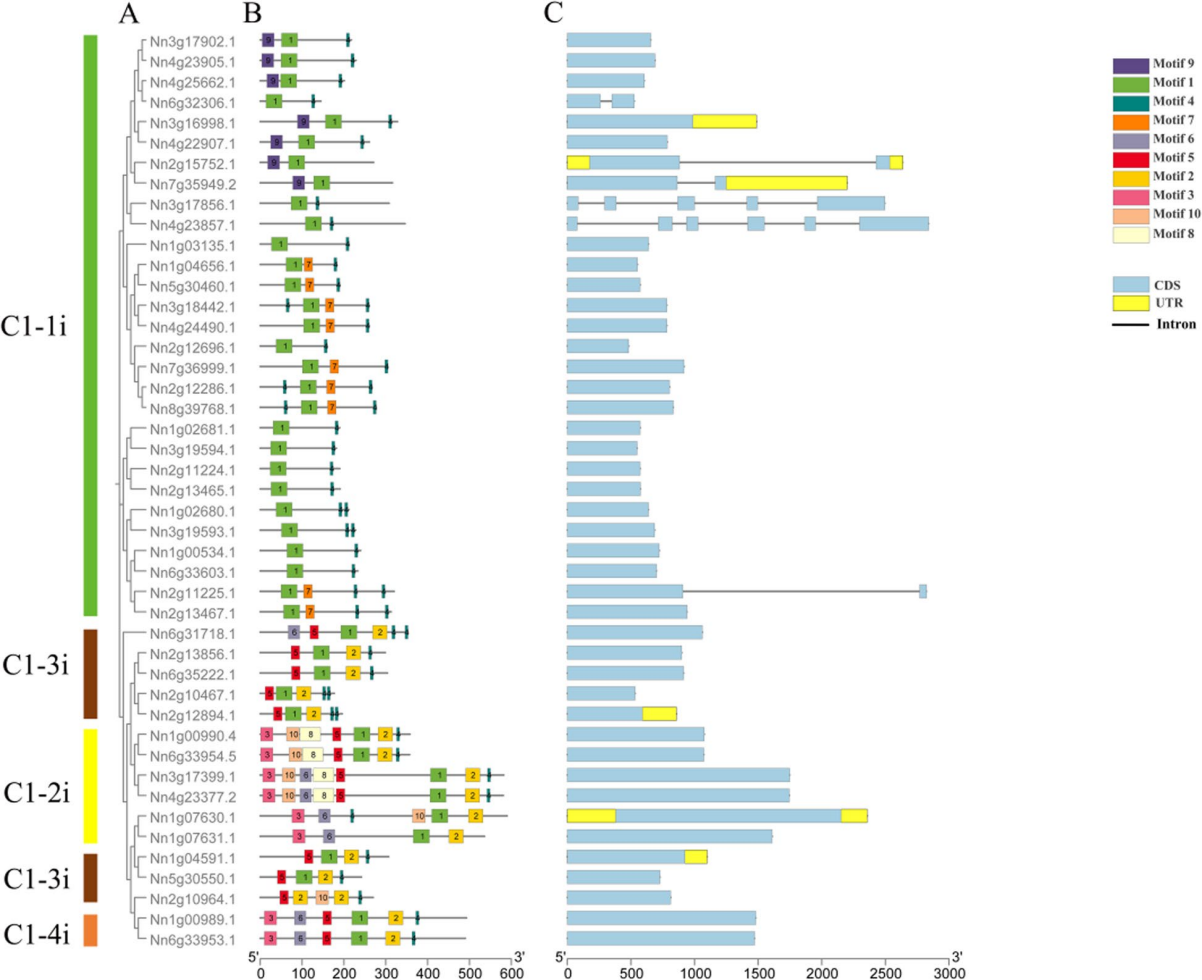
Phylogenetic relationship, conserved motifs, and exon/intron structures of Q-type *NnZFP* genes. **A** Multiple alignment of 45 full-length amino acid sequences of Q-type *NnZFP* genes executed by ClustalW. The phylogenetic tree was constructed using MEGA 11.0 with the maximum likelihood method. **B** Schematic representation of the conserved motifs identified by MEME 5.5.0. Each colored box represents a motif and black lines represent non-conserved sequences. **C** Exon/intron structures of Q-type *NnZFPs.* Exon/intron structures were analyzed by the Gene Structure Display Server. Exons/introns of each subgroup are represented by blue boxes and black lines

The protein motifs are highly conserved amino acid residues, which are considered to possibly have functional and structural roles in active proteins. To reveal the diversification of the identified Q-type *NnZFPs*, their full-length protein sequences were inspected for 10 conserved motifs according to the phylogenetic tree (Fig. 2A). As shown in Fig. 2B, motif 1 and motif 4 were widely discovered in almost all Q-type *NnZFPs*. Moreover, other motifs were specific to specific groups. For example, members of the C1-1i commonly contained motif7 and motif9. In the C1-2i group, certain members possessed motif2 and motif5. Groups C1-3i and C1-4i displayed a similar motif composition, including motif3, motif6, motif8 and motif10.

The evolutionary process could be forecasted by analyzing the gene structures. In order to have deep insight into gene structures of these Q-type *NnZFPs*, we analyzed the number of exons and introns. The results showed that the number of introns varied from 0 to 5. There were 39 members (86.7%) without introns, and they all belonged to the C1-2i, C1-3i and C1-4i groups. while the C1-1i group contained six members with 1–5 introns, with *Nn3g17856 and Nn4g23857* processing 4 and 5 introns, respectively (Fig. 2C). These results indicated that members within the same group of Q-type *NnZFPs* exhibited similar gene structures, but differences existed in members among different groups.

### Chromosomal distributions and gene duplications in Q-type *NnZFPs*

**Fig. 3:**
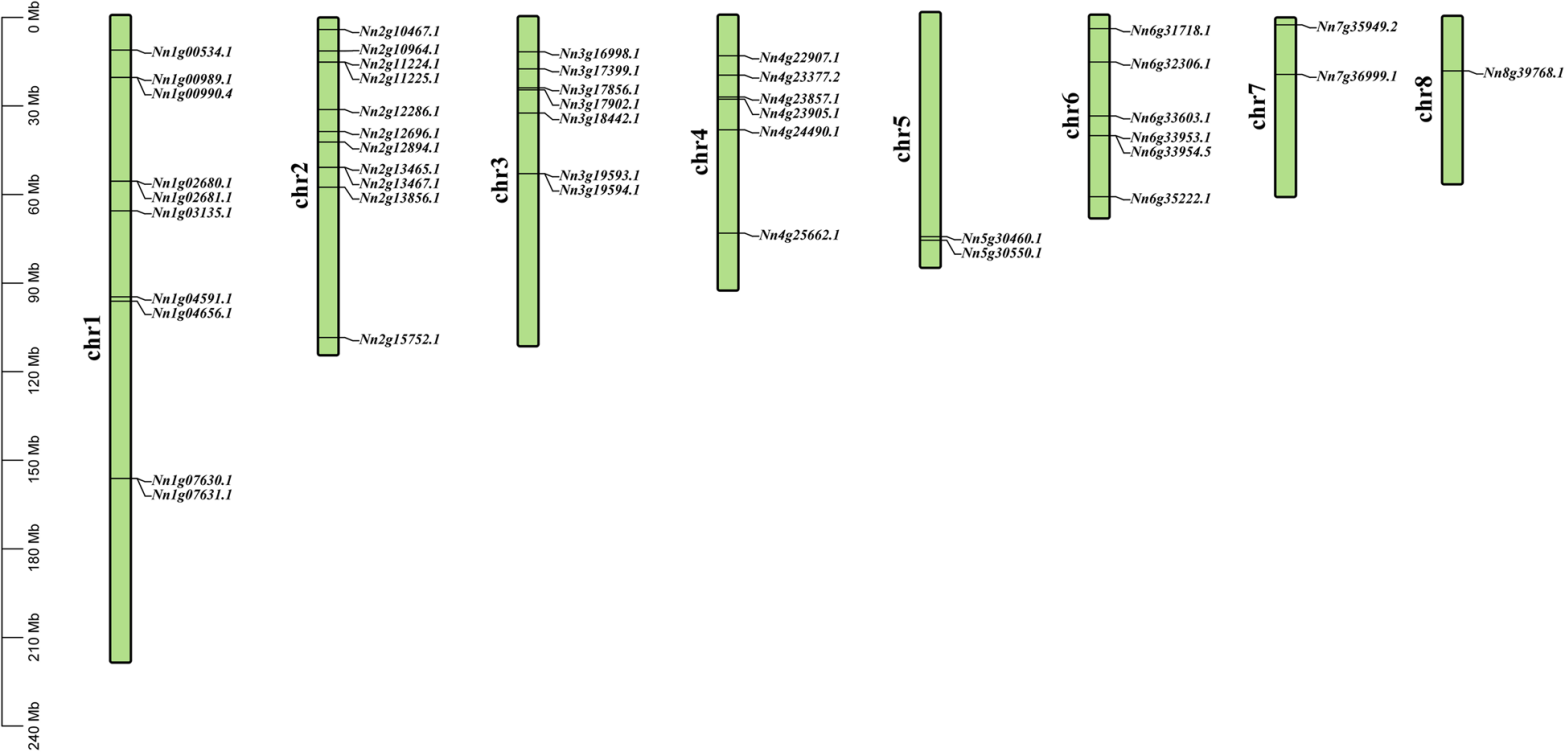
Genomic distributions of 45 Q-type *NnZFP* genes across the 8 lotus chromosomes. In the “Q-type *NnZFP* genes distribution map” of 8 lotus chromosomes, the green bars represent each chromosome, and the black lines indicate the position of each Q-type *NnZFP* gene

Members of the Q-type *NnZFPs* in lotus were unevenly distributed across 8 chromosomes. Among them, chromosome 2 had the highest number of genes, with a total of 11 genes, while only 1 gene was located on chromosome 8 (Fig. 3).

**Fig. 4:**
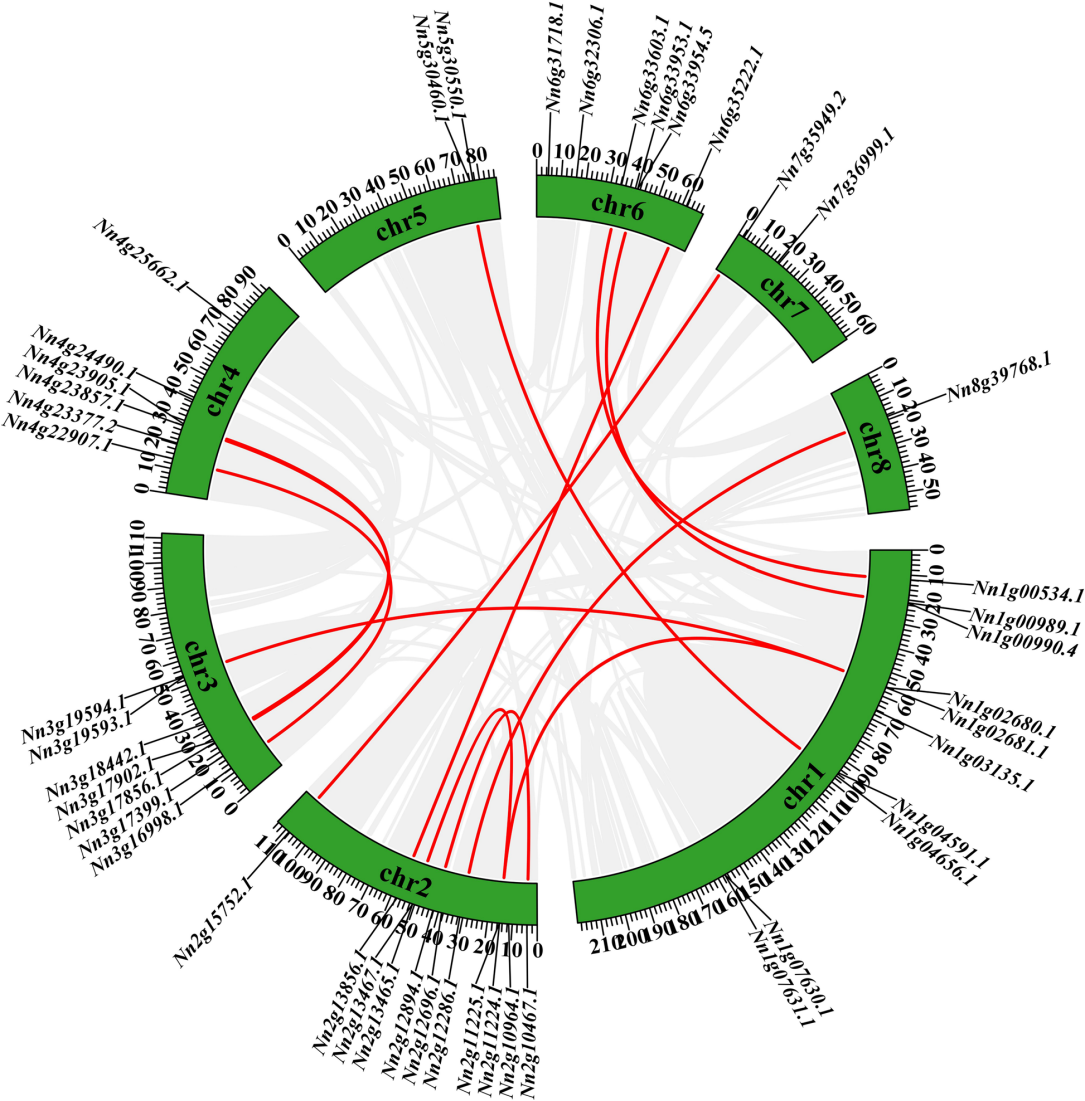
Synteny analysis of Q-type *NnZFP* genes in lotus. Grey lines represent all synteny blocks in the lotus genome. Red lines indicate the duplicated Q-type *NnZFP* gene pairs in lotus

Gene duplication lead to generate a large number of novel genes. In the present study, no tandem duplication gene pairs and 14 segmental duplication gene pairs (26/45,57.78%) were identified using MCScanX methods (Fig. 4). Among them, *Nn1g02680* and *Nn2g11224* were both collinear with two genes. In addition, we calculated that the ratio of Ka/Ks of Q-type *NnZFP* duplication gene pairs varied from 0.0849 to 0.3465 (Table S3).

**Fig. 5:**
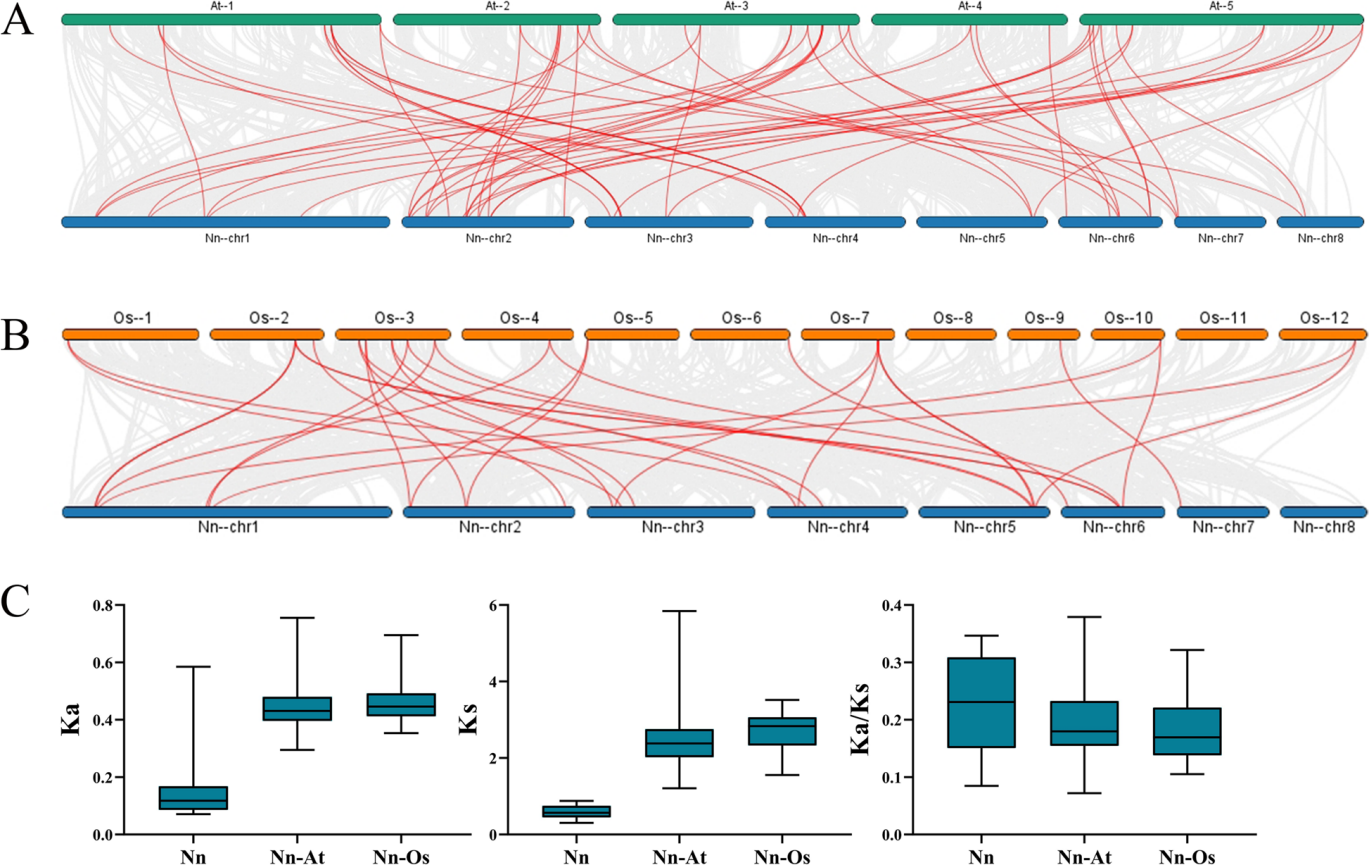
Comparative physical mapping showing the orthologous relationships of Q-type *NnZFP* genes with (**A**) Arabidopsis and (**B**) rice. The red line represents gene pairs that are homoeologous. **C**  Ka, Ks, and Ka/Ks values of homoeologous gene pairs among species

To further understand the evolutionary relationship of Q-type *NnZFPs*, we analyzed the synteny relationship of Q-type ZFP genes among lotus, Arabidopsis, and rice. The results showed that a total of 54 pairs of orthologs were identified between lotus and Arabidopsis (Fig. 5A), and the Ka/Ks ranged from 0.0720 to 0.3791 (Fig. 5C, Table S4). Furthermore, 28 pairs of orthologs between lotus and rice were identified (Fig. 5B), with the Ka/Ks varied from 0.1051 to 0.3216 (Fig. 5C, Table S5). In addition, the results indicated that 16 and 8 Q-type *NnZFP* genes were homoeologous to multiple genes in Arabidopsis and rice, respectively. Thus, the results suggested that the Q-type *NnZFP* genes in lotus had higher similarity with Q-type *AtZFP* genes in Arabidopsis.

### Analyses of Q-type *NnZFP* gene promoter *cis*-regulatory elements

*Cis*-regulatory elements play key roles in the transcriptional regulation for precise initiation and efficiency of genes. To better understand the regulatory network of Q-type *NnZFP* genes, we analyzed the promoter regions of Q-type *NnZFP* genes. And 8 *cis*-elements (AuXREs, MBSs, ABREs, LREs, JAREs, DRE/CRT, TCA-elements, WRE and WUN-motif.) that are most related to abiotic stress, hormones and plant growth were explored. According to the Figure S2 and Table S6, MBSs were widely distributed in the promoters of most of Q-type *NnZFP* genes. Considering MYB as key TFs involved in regulatory networks controlling plant development and responses to abiotic stresses, the large distribution of MBSs might enhance the role of the Q-type *NnZFP* genes in regulating the growth and development of the lotus and coping with the external environment stresses. In addition, 7 Q-type *NnZFP* genes (e.g.*Nn1g04591*, *Nn2g10467*, etc.) have a large number of ABREs in their promoters, so we speculated that these genes have similar functions with ABA-responsive factors. Taken together, our results suggested that these Q-type *NnZFP* genes may respond to different pathways through different types of *cis*-acting elements within their promoter regions.

### Analyses of tissue specific expression of Q-type *NnZFP* genes

**Fig. 6:**
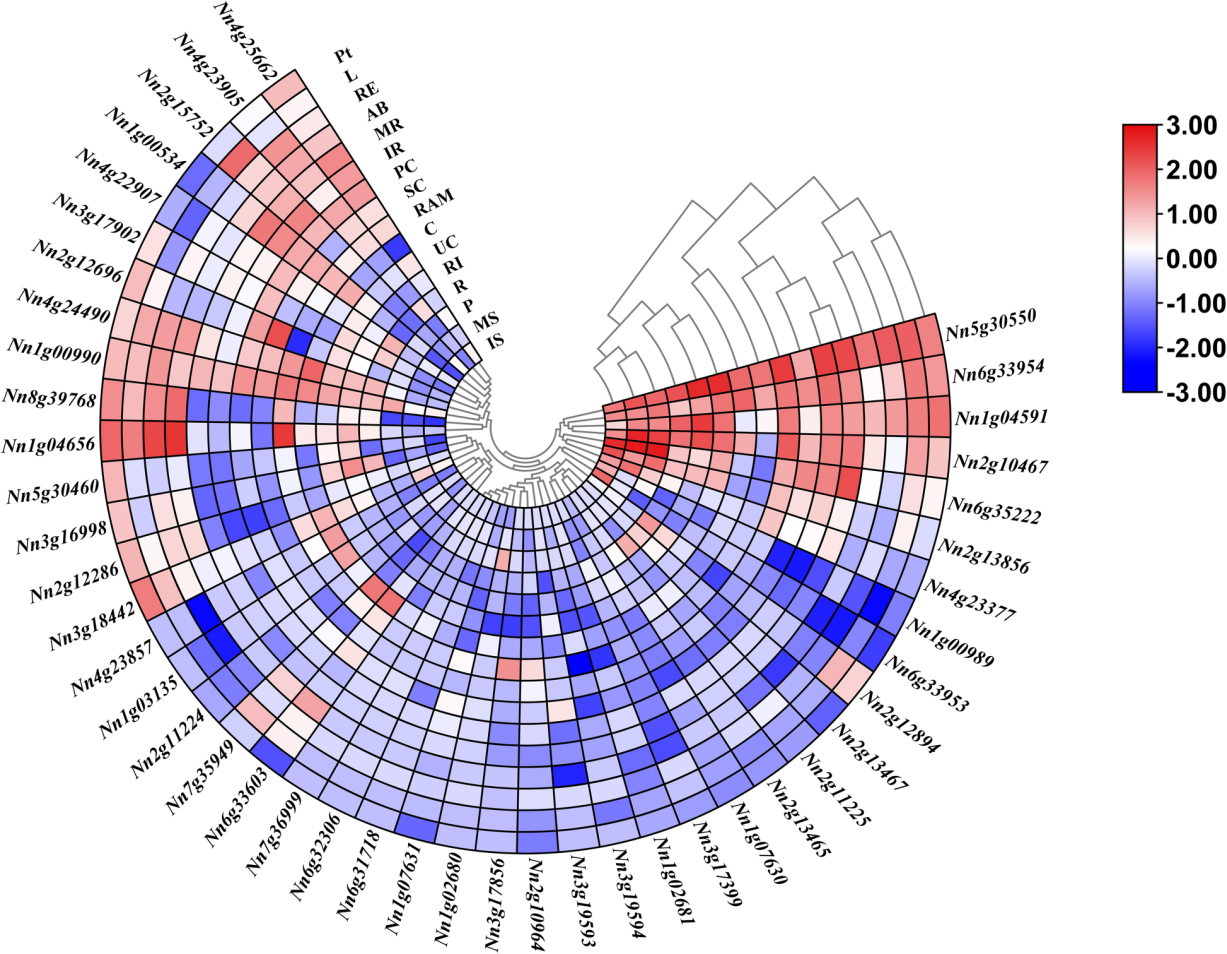
Heatmap representation of the expression patterns of Q-type *NnZFPs* among different tissues from all developmental stages. Lower and higher levels of transcript accumulation are indicated by red and blue, respectively, and the median level is indicated by white. Note: AB: Apical Bud; SC: Seed Coat; C: Cotyledon, R: Root; RI: Rhizome Internode; IR: Immature Receptacle; MR: Mature Receptacle; L: Leaf; IS: Immature Stamen; MS: Mature Stamen; P: Petal; Pt: Petiole; PC: Pollinated Carpel; UC: Unpollinated Carpel; RE: Rhizome Elongation; RAM: Rhizome Apical Meristem

To understand the tissue-specific expression patterns of the Q-type *NnZFP* genes, the transcript abundances in different tissues from all developmental stages (apical bud, seed coat, cotyledon, root, rhizome internode, immature receptacle, mature receptacle, leaf, immature stamen, mature stamen, petal, petiole, pollinated carpel, unpollinated carpel, rhizome elongation and rhizome apical meristem) of lotus [31] were analyzed (Fig. 6). The results showed that 30 (66.7%) Q-type *NnZFP* genes were expressed in at least one lotus tissue. Among them, *Nn5g30550* showed relatively high expression levels in all tested tissues with log_2_FPKM > 1, indicating that *Nn5g30550* played an essential role in lotus growth. Additionally, some Q-type *NnZFP* genes performed a tissue-specific expression pattern. For example, *Nn2g12696* was only highly expressed in seed coats, and *Nn1g04656* exhibited high expression levels in apical bud and rhizome. *Nn2g10467*, *Nn1g04591*, *Nn6g33954*, and *Nn6g35222* were highly expressed during petal development.

### Expression analyses of Q-type *NnZFP* genes in lotus under abiotic stresses

To probe the transcript abundance patterns of Q-type *NnZFP* genes under cold, drought, salt and heavy metal stresses, 12 genes were randomly selected with at least one gene from each phylogenetic clade. And the expression of the selected genes were investigated by qRT-PCR.

**Fig. 7:**
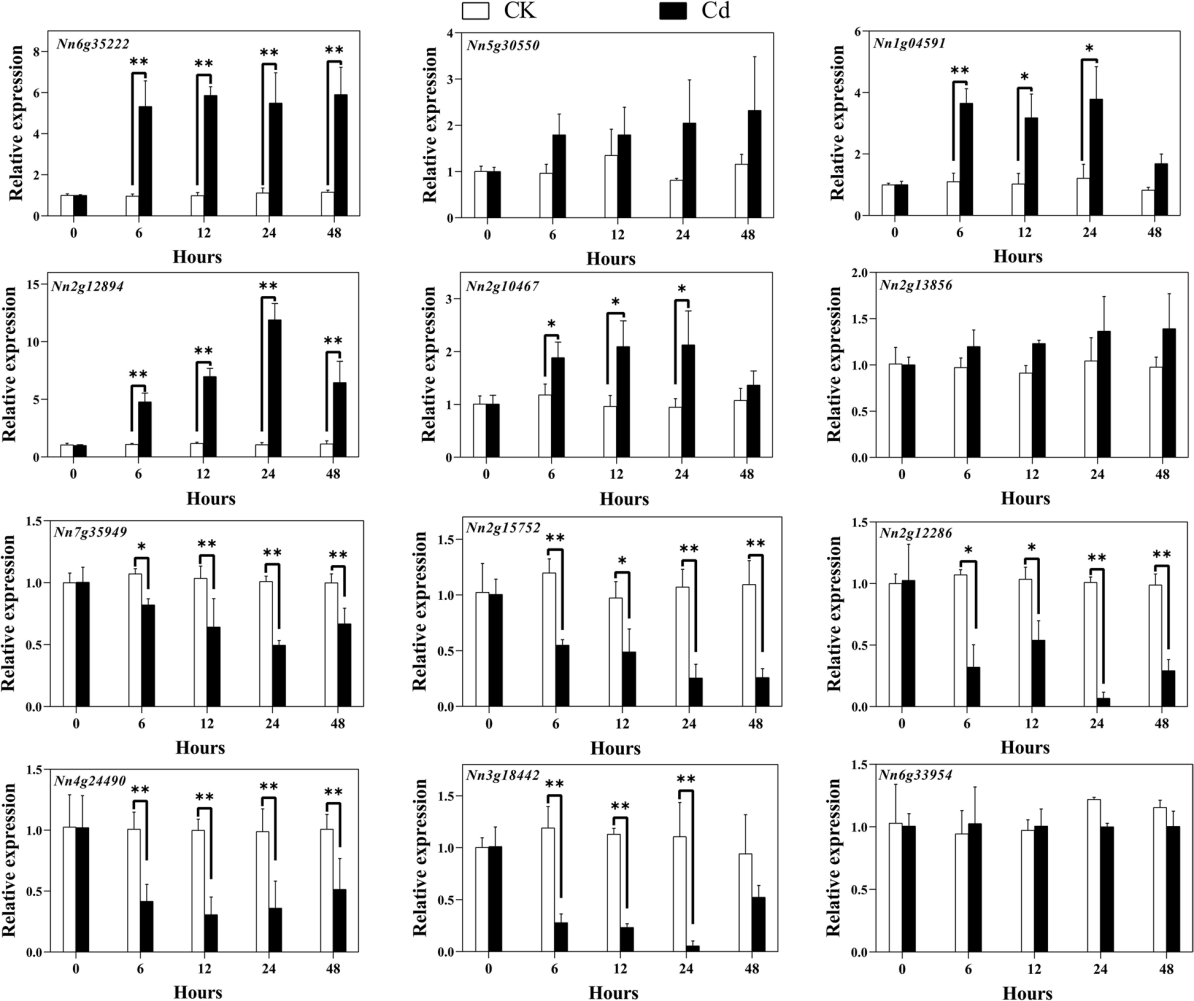
Expression analysis of Q-type *NnZFP* genes in lotus under Cd treatment, revealed by qRT-PCR. Shown are means ± standard deviations for three biological replicates and three technical replicates of each biological replicate (*: *p<*0.05; **: *p<*0.01)

Under Cd stress, 12 Q-type *NnZFP* genes showed different responses (Fig. 7). 4 genes were highly expressed under Cd stress, including *Nn6g35222*, *Nn1g04591*, *Nn2g12894*, and *Nn2g10467*. Among them, *Nn6g35222* and *Nn1g04591* expression levels immediately increased and remained relatively high levels compared with the control after 6 h of Cd treatment. By contrast, the expression levels of *Nn2g12894* and *Nn2g10467* gradually increased and reached the maximum at 24 h before a gradual decrease. Notably, the largest increase in expression was detected for *Nn2g12894*, which was 12 times more than the control. 5 Q-type *NnZFP* (*Nn7g35949*, *Nn2g15752*, *Nn2g12286*, *Nn4g24490*, and *Nn3g18442*) transcripts were down-regulated by Cd stress since 6 h. Different from the above-metioned genes, *Nn5g30550*, *Nn2g13856*, and *Nn6g33594* showed no significant changes in expression between CK and Cd treatment.

**Fig. 8:**
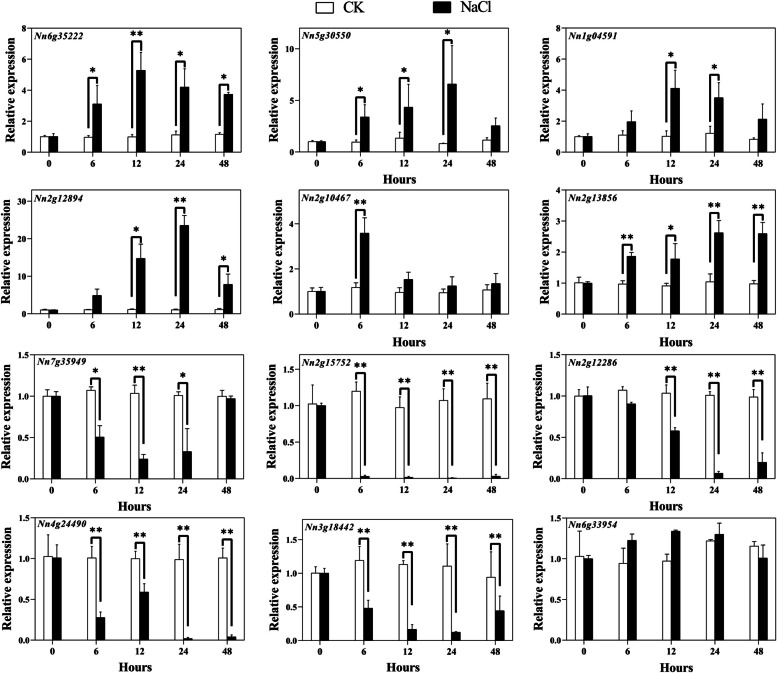
Expression analysis of Q-type *NnZFP* genes in lotus under NaCl treatment, revealed by qRT-PCR. Shown are means ± standard deviations for three biological replicates and three technical replicates of each biological replicate (*: *p<*0.05; **: *p<*0.01)

The changes of Q-type *NnZFP* gene expression under salt stress can be divided into three situations (Fig. 8). Firstly, compared with CK, six genes were induced by salt stress, including *Nn6g35222*, *Nn5g30550*, *Nn2g13856*, *Nn1g04591*, *Nn2g12894*, and *Nn2g10467*. Among them, *Nn2g12894* exhibited the largest increase (23-fold) in expression at 24 h by salt stress. Secondly, the expression levels of 5 genes (*Nn7g35949*, *Nn2g15752*, *Nn2g12286*, *Nn4g24490*, and *Nn3g18442*) were lowered under NaCl treatment than those in CK. Lastly, *Nn6g33594* maintained relatively stable expression levels after salt treatment compared with control, similar to its expression patterns under Cd stress.

**Fig. 9:**
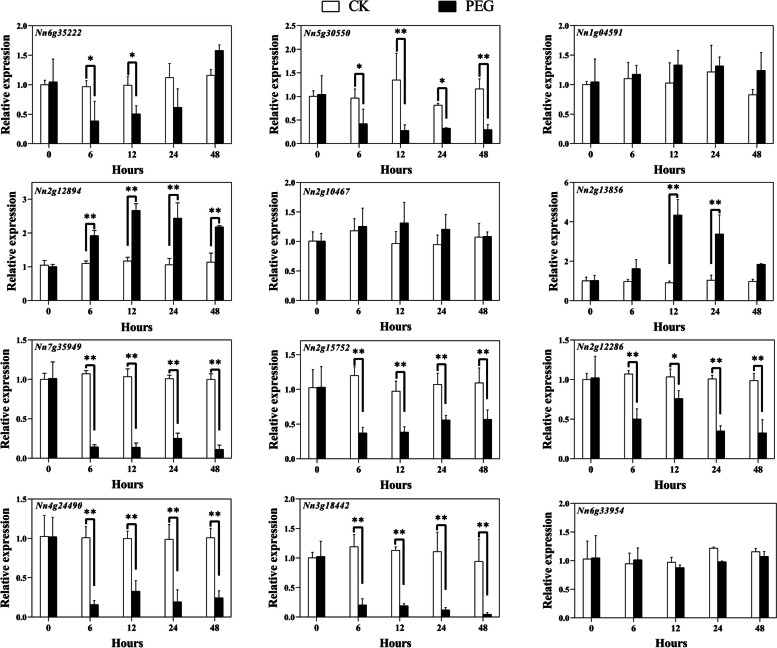
Expression analysis of Q-type *NnZFP* genes in lotus under PEG treatment, revealed by qRT-PCR. Shown are means ± standard deviations for three biological replicates and three technical replicates of each biological replicate (*: *p<*0.05; **: *p<*0.01)

For the drought treatment, two genes (*Nn2g13856* and *Nn2g12894*) were significantly up-regulated compared to CK (Fig. 9). They both reached their maximum expression levels at 12 h with 2.8 and 4.3 times that of the control, respectively. Compared to CK, expression levels of 7 genes (*Nn6g35222*, *Nn5g30550*, *Nn7g35949*, *Nn2g15752*, *Nn2g12286*, *Nn4g24490*, and *Nn3g18442*) were down-regulated under drought treatment. In addition, after PEG treatment, there were three genes with stable expression levels similar to CK, including *Nn1g04591*, *Nn2g10467*, and *Nn6g33594*.

**Fig. 10:**
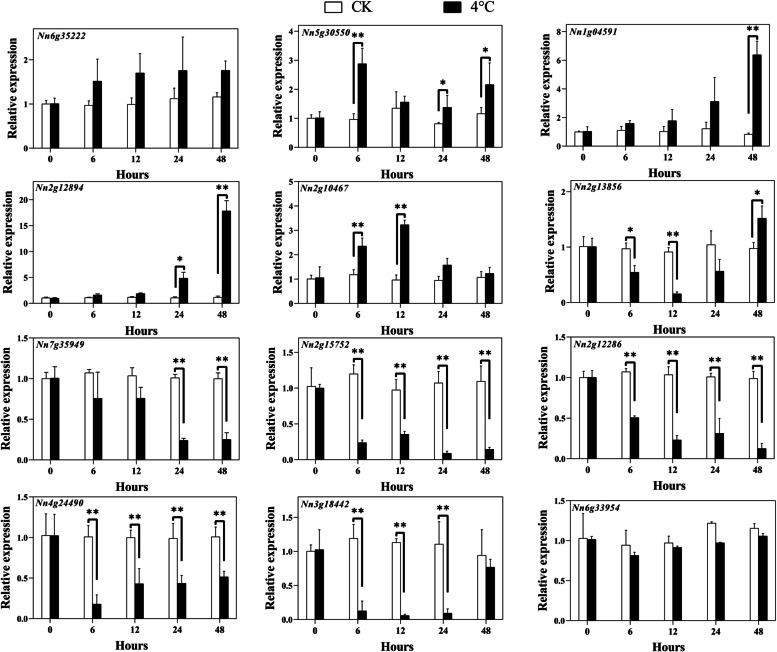
Expression analysis of Q-type *NnZFP* genes in lotus under 4℃ treatment, revealed by qRT-PCR. Shown are means ± standard deviations for three biological replicates and three technical replicates of each biological replicate (*: *p<*0.05; **: *p<*0.01)

For low-temperature treatment, the results revealed that transcripts of four genes (*Nn5g30550*, *Nn1g04591*, *Nn2g12894*, and *Nn2g10467*) were up-regulated (Fig. 10). The highest expression was observed for *Nn2g12894*, which was 17.3 times more than control. In contrast, *Nn2g13856*, *Nn7g35949*, *Nn2g15752*, *Nn2g12286*, *Nn4g24490*, and *Nn3g18442* were down-regulated under low-temperature treatment. Compared to CK, 2 genes, named *Nn6g35222* and *Nn6g33594*, showed no evident changes in expression under cold stress.

It’s worth noting that out of 12 Q-type *NnZFP* genes, *Nn2g12894* was induced by all four stresses treatments, and another 10 genes including *Nn6g35222*, *Nn2g13856*, *Nn7g35949*, *Nn2g15752*, *Nn2g12286*, *Nn4g24490*, *Nn3g18442*, *Nn5g30550*, *Nn1g04591*, and *Nn2g10467* responded to three different stress treatments. However, *Nn6g33594* showed little responses to any abiotic stresses in this study.

## Discussion

TFIIIA-type ZFPs, known as C2H2-ZFPs, were firstly reported in the last century during the study of amphibian oocyte cells [3]. Q-type ZFPs are a subfamily of C2H2-ZFPs and play essential roles in plant growth and development, and responses to abiotic stresses. To date, Q-type ZFP gene family has been identified in many species. For example, there are 56 members in Arabidopsis [3], 47 members in wheat [34], 25 members in cabbage [35], and 58 members in alfalfa [36]. Similarly, a total of 45 Q-type *NnZFPs* were identified through lotus genome in this study. The genome size of the five plants was different, with 125 Mb in Arabidopsis [37], 17 Gb in wheat [38], 530 Mb in cabbage [39], 3.15 Gb in alfalfa [40] and 929 Mb in lotus [31]. Thus, the number of Q-type ZFP superfamily members may be relatively stable in plants and it has no absolute correlation with genome size.

The number of introns and exons can affect the functional evolution of genes [41]. Structural analyses of the Q-type ZFP subfamily showed that 86.7% of Q-type *NnZFP* genes had no introns (Fig. 2C). Low intron numbers of genes accelerate its process of transcriptional expression, and it is convenient to decrease the cost for transcription and make cell a fast reaction to abiotic stresses [42]. As a result, these Q-type *NnZFP* genes may play an important role in the corresponding stress of lotus. The motif composition analysis of Q-type *NnZFPs* revealed that all 45 members contained motif1, which was identified as the core sequence specific to Q-type *NnZFPs* (Fig. S1A) and played an important role in DNA binding [43]. Additionally, motif4, identified as the ethylene-responsive element binding-factor-associated amphiphilic repression (EAR) motif, was present in 42 out of the 45 members (93.3%) (Fig. S1B). The EAR-motif is one of the most dominant transcriptional repression motifs identified in plants. To date, many Q-type ZFPs containing the EAR-motif reported in plants are recognized as transcriptional repressors [44]. For example, the LATE FLOWERING (LATE) gene (*At5g48890*) and the KNUCKLES (KNU) (*At5g14010*) are demonstrated as transcriptional repressors of cellular proliferation in *Arabidopsis thaliana* L. [45, 46]. In addition, the EAR-motif transcription factor ZAT12 was demonstrated to be function as a negative regulator for iron (Fe) deficiency responses through its direct interaction with the bHLH protein FIT in Arabidopsis [47]. These results revealed that the Q-type *NnZFPs* are rich in potential transcriptional inhibitors. But extensive researches have also shown that some Q-type *NnZFPs* have positive regulatory effects. For example, *GsGIS3* enhances tolerance to Al toxicity through positive regulation of Al-tolerance-related genes [48], and *OsZFP15* is a positive regulator of ABA catabolism and thus accelerates seed germination [49].

Gene duplication contributes to the amplification of new gene family members [50], and some evolving new members may lose their original functions or acquire new functions to enhance plant adaptability or become pseudogenes [51]. In our research, no tandem duplication was found and 14 segmental duplication pairs (26/45, 57.78%) were identified (Fig. 4, Table S3). This result suggested that the expansion of the Q-type *NnZFP* gene family might be mainly caused by the segmental duplication. A Ka/Ks value of 1 denotes neutral selection, Ka/Ks less than 1 suggests purification selection and Ka/Ks more than 1 indicates positive selection [52]. The Ka/Ks ratios of all duplication genes pairs in this work varied from 0.0849 to 0.3465, suggesting that the Q-type *NnZFP* genes might have experienced purifying selection in the process of evolution. These duplicated Q-type *NnZFP* gene pairs were the results of natural and artificial selections during species evolution, and it is important to investigate their duplication relationship for further researches on the evolutionary relationship of this gene family in lotus.

Q-type C2H2 ZFPs are widely involved in the plant growth and development, including seed germination and maturation [53], leaf development [15], and floral organ development [17]. In the present study, *Nn5g30550* was observed to be preferentially expressed in all tested organs (Fig. 6), suggesting a key role of this gene in regulating lotus growth and development. Two genes named *AtAZF1* and *AtAZF2*, homoeologous to *Nn5g30550* (Table S4), were demonstrated to affect the expression of many ABA-repressive genes in plants and severely affected seedling growth [54]. Additionally, some Q-type *NnZFP* genes performed a tissue-specific expression pattern. For example, *Nn6g35222* was highly expressed during petal development (Fig. 6), indicating that it may be associated with floral organ development. It has been reported that *At3g10470*, which was homoeologous to *Nn6g35222* (Table S4), affected male gametophyte development in Arabidopsis [55]. Furthermore, *Nn2g12696* was only highly expressed in seed coats (Fig. 6). The phylogenetic analyses showed *Nn2g12696* was clustered with *AtZFP3* (*At5g25160*), which could influence seed germination by interfering with ABA and light signaling [56]. These results may lead to more directed understanding the function of these Q-type *NnZFP* genes in lotus development biology.

Plant growth is frequently threatened by environmental stresses, such as drought, low temperature, salt, and heavy metals [57–60]. Many stress-related Q-type ZFPs can help plants adapt to these abiotic stresses. For instance, *GhZAT6* could resist salt stress in *G. hirsutum* by regulating ROS-related gene expression [61]; *JcZAT10*, which was regulated by ZAT12 and CBFs in *Jatropha curcas*, controlled the expression of low-temperature-related COR genes, thereby enhancing its cold resistance [62]. In our study, a total of 12 Q-type *NnZFP* expression patterns under abiotic stresses were explored using qRT-PCR, and we found most of these Q-type *NnZFP* transcripts changed under different stress treatments. According to the expression profiles in Figs. 7, 4 genes were up-regulated under Cd treatment, including *Nn6g35222*, *Nn1g04591*, *Nn2g12894*, and *Nn2g10467*, suggesting the involvement of them in responses to Cd stress. To date, limited researches have focused on the role of Q-type *NnZFPs* in plant Cd tolerance. As far as we known, *AtZAT6* (*At5g04340*) is demonstrated to improve Cd tolerance in Arabidopsis through specifically binding to the promoter of *GSH1* and regulating glutathione synthesis in Arabidopsis [22], and *AtZAT10* (*At1g27730*) was demonstrated to play dual roles in cadmium uptake and detoxification in Arabidopsis [63]. Generally, members within one cluster may have common evolutionary origins and conserved functions [64]. *Nn6g35222*, *Nn1g04591*, *Nn2g12894*, and *Nn2g10467*.were clustered with *AtZAT6* and *AtZAT10* in C1-2i in our findings, indicating that they may have a positive role in Cd tolerance in lotus. Moreover, 6, 2, 4 genes were induced by salt, drought and cold stresses, respectively (Fig. 8–10). Similarly, *AtZAT18* was demonstrated to be involved in drought tolerance in Arabidopsis [65], and *OfZAT35* is an important regulator of cold tolerance in transgenic tobacco [66]. We also found that there is a large overlap of Q-type *NnZFP* genes has multiple roles in three or more stresses (Fig. 7–10). For example, *Nn2g13856* and *Nn2g10467* could be induced by Cd, NaCl and PEG treatment at the same time. Specially, we found that *Nn2g12894* was induced by all the four treatments. It may be related to the fact that this gene containing multiple stress and hormone related *cis*-elements in its promoter, such as JARE, AuxRE, LREs, CBF and MBSs. These results indicated that these genes carried out multiple physiological and biochemical functions to faced environmental challenges. Therefore, the expression patterns of Q-type *NnZFP* genes under various abiotic stresses provided many new candidate genes for further exploration of the mechanisms of resistance in lotus.

## Conclusion

In the current research, we performed a comprehensive analysis of Q-type *NnZFPs* family in lotus. A total of 45 Q-type *NnZFPs* were identified in the lotus genome and unevenly mapped on 8 chromosomes. These Q-type *NnZFPs* were divided into four groups, including C1-1i, C1-2i, C1-3i and C1-4i. Segmental duplication events played a key role in the expansion of the Q-type *NnZFP* gene family and these gene pairs evolved under strong purifying selection. Synteny analyses indicated that 54 and 28 *NnZFP* genes were orthologous to Arabidopsis and rice, respectively, suggesting that the Q-type *NnZFP* genes have higher similarity with Q-type *AtZFP* genes. In addition, their expression profiles in various tissues and their development stages and responses to Cd, NaCl, drought and cold stress conditions demonstrated that this gene family was widely involved in lotus organ development and abiotic stress responses. All of these results will help to reveal the biological functions of Q-type *NnZFP* genes, and provide a basis direct for further functional analysis of these genes in growth and abiotic stress tolerance in lotus.

### Supplementary Information

Supplementary Material 1.

Supplementary Material 2.

Supplementary Material 3.

Supplementary Material 4.

Supplementary Material 5.

Supplementary Material 6.

Supplementary Material 7.

## Data Availability

The protein sequences of the Q-type C2H2 ZFP gene family in Arabidopsis thaliana L. were downloaded from database (https://www.arabidopsis.org/). The whole genome sequence data and genome annotation data of lotus were all from the lotus Genome Database (http://nelumbo.biocloud.net). The expression data of Q-type NnZFP genes in various tissues were all downloaded from the lotus Genome Database (http://nelumbo.cngb.org/nelumbo/tools/expressionVisualization).
