# Supplementary material for: Genome-wide identification of the Q-type C2H2 zinc finger protein gene family and expression analysis under abiotic stress in lotus (Nelumbo nucifera G.)
Source: BMC Genomics. 2024 Jun 28;25:648. doi: 10.1186/s12864-024-10546-1 (PMC11214253; doi:10.1186/s12864-024-10546-1)
Supplement: Supplementary file 1 — Supplementary Material 1. [file 12864_2024_10546_MOESM1_ESM.docx]

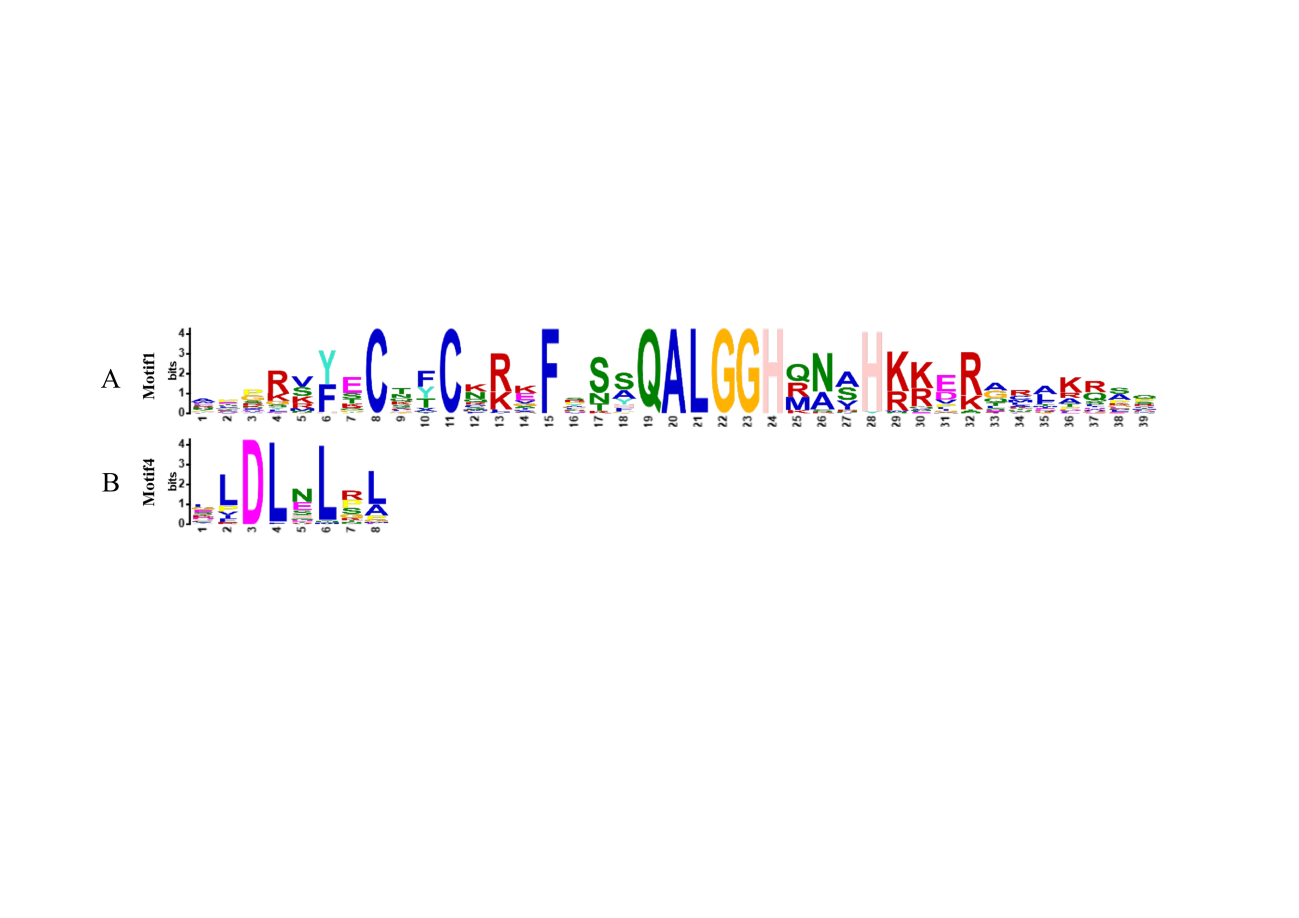


Fig. S1 Main conserved motif of Q-type *NnZFP* genes in lotus.


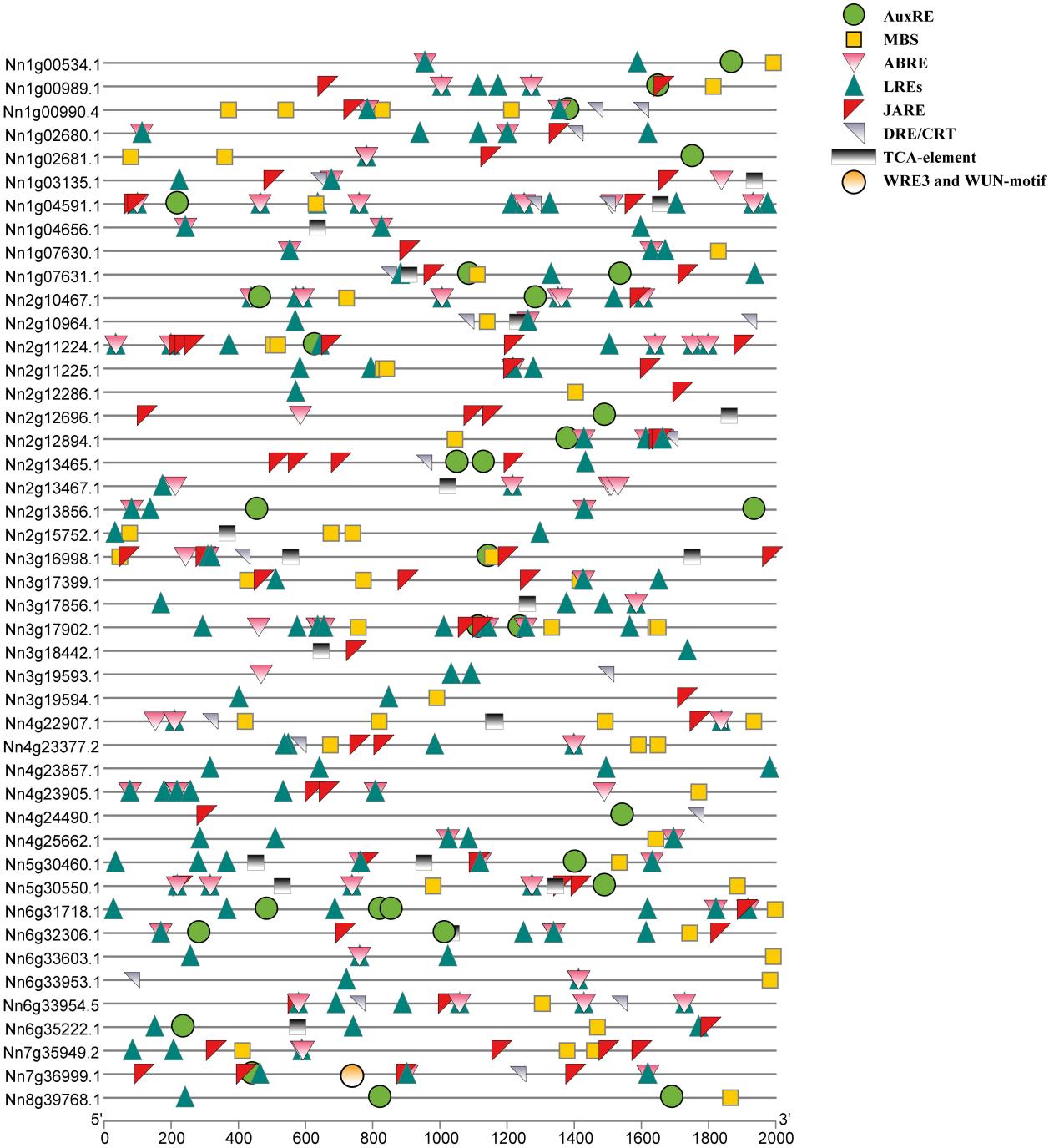


Fig. S2 Analysis of Q-type *NnZFP* Gene Promoter *Cis*-Regulatory Elements. The differently coloured patterns represent the different regulatory elements.
